# Mast-Cell Response to *Leishmania mexicana* and Sand-Fly Salivary Proteins Is Modulated by Orchiectomy

**DOI:** 10.3390/pathogens11040398

**Published:** 2022-03-25

**Authors:** Laura Sánchez-García, Armando Pérez-Torres, Samira Muñoz-Cruz, Norma Salaiza-Suazo, Jorge Morales-Montor, Ingeborg Becker

**Affiliations:** 1 Unidad de Investigación en Medicina Experimental, Centro de Medicina Tropical, Facultad de Medicina, Universidad Nacional Autónoma de México, Ciudad de México 04510, Mexico; salsua@unam.mx (N.S.-S.); becker@unam.mx (I.B.); 2 Departamento de Biología Celular y Tisular, Facultad de Medicina, Universidad Nacional Autónoma de México, Ciudad de México 04510, Mexico; armandop@unam.mx; 3 Unidad de Investigación Médica en Enfermedades Infecciosas y Parasitarias, Centro Médico Nacional Siglo XXI, Instituto Mexicano del Seguro Social, Ciudad de México 06720, Mexico; mcsamira@unam.mx; 4 Departamento de Inmunología, Instituto de Investigaciones Biomédicas, Universidad Nacional Autónoma de México, Ciudad de México 04510, Mexico; jmontor66@biomedicas.unam.mx

**Keywords:** mast cells, *Leishmania mexicana*, sand fly salivary proteins, sexual hormones

## Abstract

Mast cells (MCs) play a crucial role during *Leishmania* infections, which is transmitted through the bite of an infected sand fly that injects saliva together with the parasite. Sand fly saliva is a complex fluid that modulates the host immune response. In addition, hormonal factors modulate the host immune response and alter susceptibility to infections. Thus, to assess the impact of male sex hormones on the mast-cell (MC) response to *Leishmania* infections, we orchiectomized male mice, infected them with the parasite in the presence of sand fly salivary proteins, and analyzed the inflammatory response of MCs. Our results showed that the MC response to the parasite and vector salivary proteins differed between orchiectomized and sham-operated mice. In orchiectomized mice, MC showed a retarded activation pattern, associated with slower degranulation and weaker TNF-α, histamine, and tryptase staining in response to the infection with *Leishmania mexicana* combined with vector-salivary proteins, as compared to sham mice. Furthermore, neutrophil infiltration was slower in orchiectomized mice, and numbers of infected macrophages and lesion sizes were smaller. Our results show that, during *Leishmania* infection, male sex hormones modulate the mast-cell response against the parasite and salivary proteins of the sand fly vector, inducing an intense inflammatory response. Their absence in orchiectomized mice retards the inflammatory response, enabling better control of the infection and slower disease progression.

## 1. Introduction

Mast cells (MCs) are sentinels of innate host defense due to their ability to detect pathogens and immediately degranulate upon activation. They are strategically distributed throughout most tissue types of the body and can differentiate into lifelong sessile tissue homing cells [1,2,3], making them unique. Mast cells mediate their physiological and pathophysiological roles through the selective release of various preformed and newly synthesized inflammatory mediators [4,5,6]. These include different proteases, histamine, heparin proteoglycan, chondroitin sulfate E, acidic hydrolases, and various cytokines and growth factors [4,5,6]. However, the role of activated MCs in response to infections remains controversial and is a double-edged sword: on the one hand, specific mediators can contribute to containing or eliminating the pathogen, yet they can also favor the progression of infections by modulating the intensity of inflammation depending on the pathogen, age, gender, and the genetic background of the host [4,5]. *Leishmania* infections (both in vitro and in vivo) showed that MC degranulation influences the early inflammatory response, thereby contributing to the outcome of infection, which also depends on the genetic background of the host [7,8,9]. Thus, in BALB/c mice, MCs facilitate disease progression, while in C57BL/6 mice, MCs promote disease control [7]. *Leishmania* transmission occurs through the bite of infected female sand flies during blood feeding, where they inject the parasite together with salivary components [10,11], both of which affecting the immune response of the host [12]. The immune system is also strongly influenced by the endocrine system, since sex hormones modulate and regulate the differentiation, proliferation, and activation of immune cells [13,14]. During *Leishmania* infections, the gender of the host modulates the immune response [15,16,17,18,19]. Thus, estrogens upregulate IL-4 and IL-10 production, whereas testosterone downregulates IL-2, IL-6, TNF-α, and nitric oxide production in macrophages [20,21,22]. During *L. donovani* infections, testosterone downregulates p38 MAPK activation, inhibiting macrophage inflammatory and microbicidal responses [20,21,22,23,24,25]. Additionally, dihydrotestosterone (DHT) increases *Leishmania mexicana (L. mexicana)* infectivity, both in vivo and in vitro [26].

However, the regulation exerted by male sex hormones, parasites, and sand fly salivary proteins on MCs during acute and chronic *L. mexicana* infections remains to be analyzed. Therefore, the aim of the current study was to analyze MC activation in male BALB/c mice deprived of gonadal hormones and infected with *L. mexicana* in the presence of sand-fly salivary-gland proteins.

## 2. Results

### 2.1. Ear Tissue Histology after Inoculation of L. mexicana and Vector Salivary Proteins

**Figure 1:**
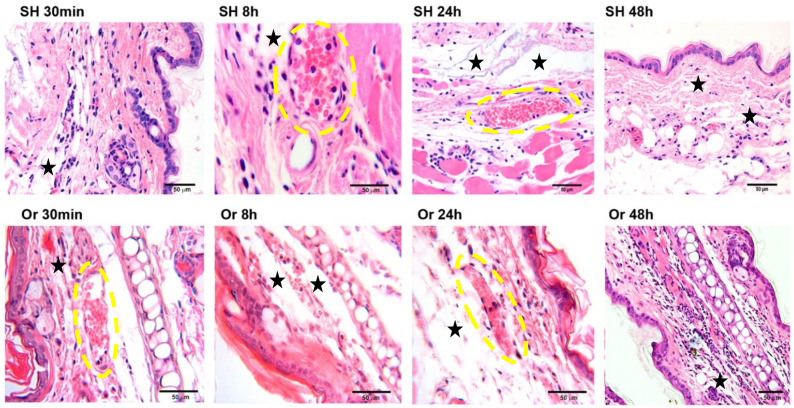
Kinetics of inflammatory cell infiltration in pinna skin from sham (SH) and orchiectomized (Or) BALB/c mice in response to intradermal inoculation of *L. mexicana* together with salivary-gland lysates. In sham mice, the dermal accumulation of inflammatory cells, principally neutrophils, was evident at 30 min after challenge (SH 30 min), and remained intense up to 48 h (SH 48 h). In contrast, in orchiectomized mice, dermal neutrophil infiltration was delayed, beginning at 24 h after challenge and remaining intense throughout 48 h (Or 48 h). Neutrophils were identified by their multilobed nuclei typically consisting of 3 to 5 segments. Yellow ovals show vasodilation and vascular congestion, and black stars show edema. Photomicrographs are representative of three independent experiments with five replicates for each group. Hematoxylin–eosin staining was used. Scale bars are of different sizes because photomicrographs had small magnification differences due to the use of optovar.

To assess MC activation in the presence or absence of male sexual hormones after *L. mexicana* and salivary-gland lysate inoculation, the ears of intact, sham, and orchiectomized BALB/c male mice were analyzed by histology. After the inoculation of *L. mexicana* and vector salivary proteins, intact BALB/c male mice showed a dermal inflammatory response initiating at 30 min after the challenge and continuing throughout 8 to 72 h, during which neutrophilia ensued (Figure 1). Orchiectomized mice also showed edema, vascular congestion, and vasodilatation, yet with lower neutrophilia and dermal neutrophil infiltration as compared to intact mice between 30 min and 24 h after inoculation. Interestingly, a delay of 48 h was observed in the recruitment of neutrophils in orchiectomized mice (Figure 1).

**Figure 2:**
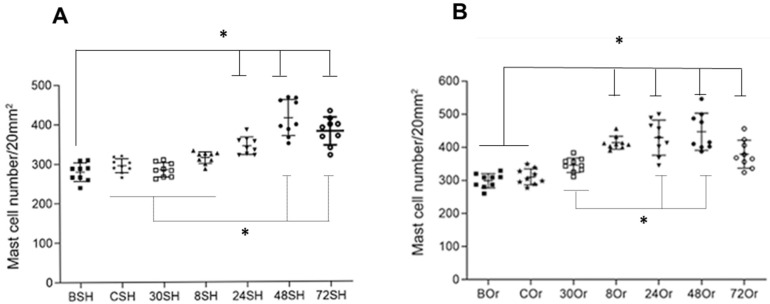
Mast-cell (MC) numbers from pinna skin samples of sham (SH) and orchiectomized (Or) mice after intradermal inoculation of *L. mexicana* with salivary gland lysates. Toluidine blue-stained MCs counted in 20 mm^2^ of tissue sections f rom (**A**) sham-operated and (**B**) orchiectomized mice. In sham-operated mice (**A**,*), a significantly lower number of MCs were found in tissues in basal conditions (BSH), as compared to at 24, 48, and 72 h postinfection (24SH, 48SH, 72SH). CSH, 30SH and 8SH were significantly lower as compared to 24SH and 48SH. In orchiectomized mice (**B**,*), significantly lower numbers of MCs were found in basal and control conditions (BOr and Cor), as compared to 8, 24, 48 and 72 h post infection (8Or, 24Or, 48Or and 72OR). At 30 min (30Or) post infection, the MC numbers were significantly lower as compared to 24 and 48 h (24Or, 48Or) in orchiectomized animals. Nonparametric ANOVA ordinary multiple comparisons was employed (Significance *p* < 0.0001). (Abbreviations: BSH: basal sham; CSH: control sham; 30SH: 30 min sham; 8SH: 8 h sham; 24SH: 24 h sham, 48SH: 48 h sham; 72SH: 72 h sham. BOr (basal orchiectomized; COr control orchiectomized; 30Or: 30 min orchiectomized, 8–72 Or: 8–72 h orchiectomized). Three independent experiments with five replicates for each group.* *p* ˂ 0.001.

Furthermore, numbers of recruited MC and their degranulation were also slightly different between orchiectomized and sham mice. A slightly higher increase in MC infiltration was observed in gonadectomized mice as compared to sham mice at all time points (Figure 2).

After *L. mexicana* and salivary gland lysate inoculations, MC recruitment started at 30 min until 48 h, showing a similar pattern between orchiectomized and sham mice (Figure 2). Sham mice showed slightly lower MC numbers throughout all time points than those of orchiectomized mice. At 72 h, no MC increase was observed in either group of mice (Figure 2). In sham mice, MC numbers significantly increased after 24 h and throughout 72 h, compared to basal values (BSH) (*p* ˂ 0.001). Furthermore, MCs increased significantly at 48 and 72 h, when comparing CSH, 30SH and 8SH (*p* ˂ 0.001). In orchiectomized mice, a significant increase in MC numbers was registered after 8 to 72 h when compared to basal and control values (BOr, COr) (*p* ˂ 0.001). Enhanced MC numbers were also observed when comparing values at 30 min (30Or) to those of 24 to 48 h (*p* ˂ 0.001) (Figure 2).

When comparing MC numbers between groups of orchiectomized vs. sham mice, significant differences were observed in MC numbers between 30 min and up to 48 h, showing that sham mice always showed lower numbers (Table 1).

**Figure 3:**
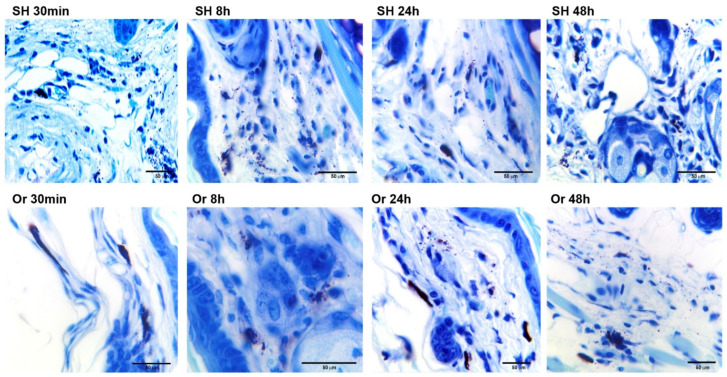
Photomicrographs of mast-cell (MCs) degranulation/activation in pinna skin from sham (SH) and orchiectomized (Or) mice after challenge with *L. mexicana*, together with salivary gland lysates. Toluidine blue-stained sections from inoculated ears showed a differential MC degranulation pattern between the groups of SH and Or mice. Overall, Or mice showed intact dark purple MCs without apparent degranulation at 30 min. At 8, 24, and 48 h, increase in released granules was observed (see Or 8, 24, and 48 h). In contrast, in SH mice, evident MC degranulation/activation was found at 30 min with dermal scattered granules. Degranulation increased at SH 8, 24, and 48 h. Images are representative of three independent experiments with five replicates for each group. Mast cells were identified by cytoplasmic granules showing metachromatic properties (alkaline toluidine blue and red–violet stain of secretory granules) and expelled granules with pink stain.

In orchiectomized animals, MC degranulation revealed a discrete group of granules at 30 min and 48 h. However, at 72 h, MC degranulation in orchiectomized animals was similar to that observed in sham mice at 30 min and 8 h (Figure 3). In orchiectomized mice, MC degranulation resembled a piecemeal process in which the contents of the released granules were surrounded by plasma membranes (Figure 3); in sham mice, the MC degranulation occurred more diffusely (systematic mode), resembling activation after FceRI high-affinity receptor binding (Figure 3).

### 2.2. Immunohistochemistry of Ears after Inoculation of L. mexicana and Vector Salivary Gland Lysates: Inflammatory Mediators Released after MC Activation

**Figure 4:**
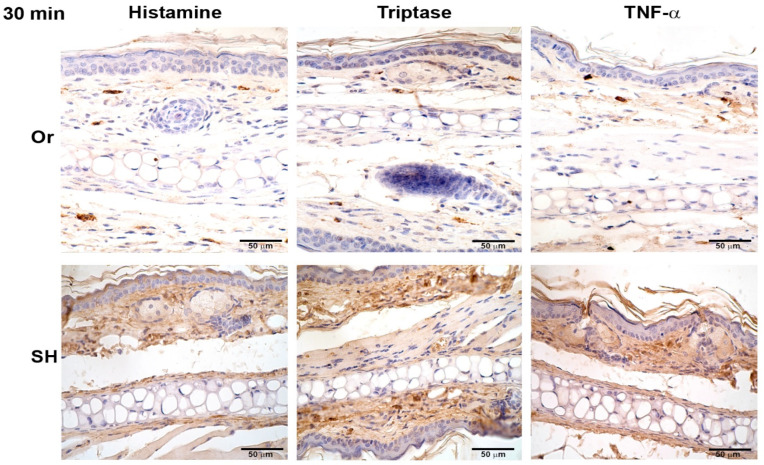
Comparative immunohistochemical assessment of histamine, tryptase, and TNF-α release in pinna skin mast cells (MCs) from sham (SH) and orchiectomized (Or) BALB/c mice, at 30 min in response to the dermal inoculation of *L. mexicana*, combined with salivary gland lysate. The immunoreactivity of inflammatory factors in MCs was more intense in Or mice than in SH mice. However, a diffuse and intense interstitial mark was observed only in SH mice. Images representative of three independent experiments with five replicates for each group.

**Figure 5:**
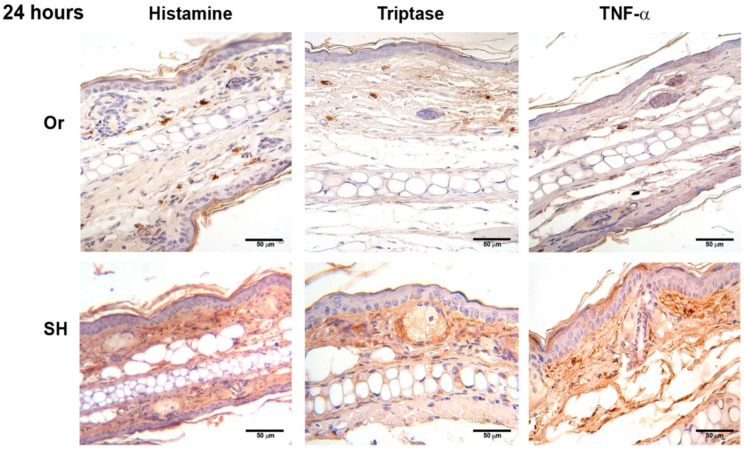
Comparative immunohistochemical assessment of histamine, tryptase, and TNF-α release in pinna skin mast cells (MCs) from sham (SH) and orchiectomized (Or) BALB/c mice, at 24 h in response to the injection of *L. mexicana*, combined with salivary gland lysate. Immunoreactivity of inflammatory factors in MCs was similar between Or and SH mice. However, diffuse and intense mark remained only in SH mice. Images representative of three independent experiments with five replicates for each group.

**Figure 6:**
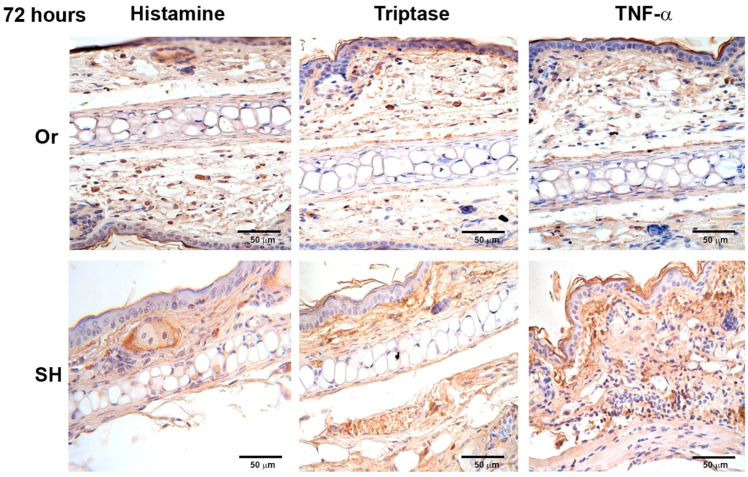
Comparative immunohistochemical assessment of release of histamine, tryptase, and TNF-α in pinna skin mast cells (MCs) from sham (SH) and orchiectomized (Or) BALB/c mice, at 72 h in response to the injection of *L. mexicana* combined with salivary gland lysate. Immunoreactivity of inflammatory factors in MCs from Or mice. Images representative of three independent experiments with five replicates for each group.

Histamine, TNF-α, and tryptase were assessed after MC activation induced by injection of *L. mexicana* and salivary gland lysates at 30 min, and 8, 24, 48, and 72 h. Inflammatory mediators released by orchiectomized mice showed differences compared to sham mice. Histamine, TNF-α, and tryptase released in orchiectomized mice at 30 min showed a weak stain, as compared with sham mice (Figure 4), which increased throughout the infection time. At 24 h, the inflammatory mediators increased in intensity but continued to be less intense than in sham mice (Figure 5). However, at 72 h, the histamine, TNF-α, and tryptase stain in orchiectomized mice increased in intensity, similar to that of sham mice (Figure 6). Therefore, the lack of male sexual hormones seemed to reduce and retard MC activation, their release of granules, and their numbers in the tissues (Figure 4, Figure 5 and Figure 6).

### 2.3. Chronic Infection of L. mexicana Together with Vector Salivary Gland Lysates in Orchiectomized BALB/c Mice

**Figure 7:**
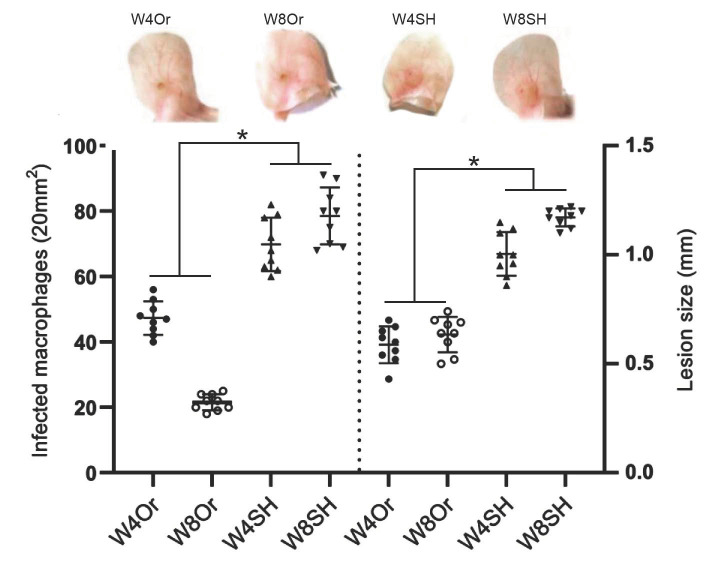
Comparative sizes of pinna skin ulcered nodules at 4 and 8 weeks (W) after *L. mexicana* promastigote infections, including counts of infected macrophages. Orchiectomized mice (Or) produced smaller lesions as compared to sham-operated (SH) mice. Lower numbers of infected macrophages were evidenced in Or mice (42.8 ± 0.2 and 21.6 ± 0.4/20 mm^2^) and smaller lesions (0.58 ± 0.084 mm and 0.62 ± 0.074 mm) after 4 or 8 weeks, respectively, as compared to SH mice (71.2 ± 0.3 and 79.8 ± 0.2/20 mm^2^) and (1 ± 0.1 mm and 1.1 ± 0.04 mm), at the same time points. Orchiectomized mice showed strict control of parasitic infection at 8 weeks. Images representative of three independent experiments with five replicates for each group. Significant differences (*) between SH mice and Or mice (*p* ≤ 0.03).

Chronic *L. mexicana* infections (4 and 8 weeks) in orchiectomized mice showed smaller ulcers than sham mice (Figure 7). Furthermore, a significant reduction in the number of infected macrophages was observed in gonadectomized mice, as compared to sham mice (Figure 7). The significant reduction in infected macrophages observed at 8 weeks (50.4%) indicated that gonadectomized mice had better control of the parasitic infection.

## 3. Discussion

The modulation of innate and adaptive immune responses by the endocrine system results in an intricate crosstalk between the different involved actors that affects the evolution of diseases. Our current work shows that male sex hormones have a strong influence on mast-cell activation and the kinetics of *L. mexicana* infections. MCs orchestrate the inflammatory process after infection with *Leishmania*. In vitro experiments showed that MCs release TNF-α, histamine, tryptase and IL-1b after contact with the parasite [27,28,29,30]. A cause–effect variation in MC numbers and in the percentage of degranulation was reported in inoculation sites after *L. major* infections [31]. Specifically, the release of TNF-α, tryptase, and histamine by MCs possibly also mediates the inflammatory process that ensues after natural sand fly bites [32].

Gender and genetic background modulate differential responses in *Leishmania* infections. Thus, susceptibility to *L. major* and *L. mexicana* infections is higher in males than that in females [16,18,19,32,33]. Furthermore, susceptible and resistant mice to *Leishmania* differ in MC degranulation: C57BL/6 males, which are slightly resistant to *L. mexicana*, respond with delayed MC degranulation, whereas highly susceptible BALB/c male mice show a vigorous immediate response [24]. These data indicated that the delay of inflammatory mediators in C57BL/6 mice favor their control of the infection.

Considering evidence on the differential response of males to *L. mexicana* infections, we now analyzed whether the early activation of MC and their release of inflammatory mediators are influenced by male sexual hormones, thereby facilitating the *L. mexicana* infection. Our infection model also included sand-fly salivary-gland lysates to simulate natural sand fly infections. Therefore, two groups of mice, orchiectomized and sham, were inoculated with *L. mexicana* in combination with sand-fly salivary-gland lysates. Our results show that, in sham BALB/c mice, early MC degranulation accompanied by a massive neutrophil infiltration was evidenced after the inoculation of the parasite together with salivary-gland lysates (Figure 2 and Figure 3). MC degranulation released inflammatory mediators such as TNF-α, histamine, and tryptase (Figure 4, Figure 5 and Figure 6), which could contribute to neutrophil recruitment, giving an advantage to the parasites. The nonprotective effect of neutrophils resembles that of a Trojan horse that shields phagocytosed parasites from extracellular immune mechanisms, permitting their replication within neutrophils. This early evasion strategy seems to facilitate infection [34,35,36]. Our data show that, in contrast to sham-operated mice, orchiectomized mice showed a discrete mode of MC degranulation with less intense neutrophil infiltration (Figure 3) accompanied by a lower release of inflammatory mediators (Figure 4, Figure 5 and Figure 6). This notorious difference in MC activation seems to be related to a slower disease evolution in *L. mexicana* infections, which was evidenced in chronic infections of orchiectomized mice that showed smaller lesions and lower numbers of infected macrophages, as compared to those of sham mice (Figure 7).

Early neutrophil recruitment during *L. mexicana* infections also blocks the protective immune response by impairing the recruitment of dendritic cells (DCs) to the infection site [34]. Inflammatory mediators released by MCs favor recruiting the early wave of neutrophils, thereby facilitating the infection. Furthermore, the release of TNF-α, histamine, and tryptase by MC contributes to creating an immune microenvironment that facilitates the establishment of *Leishmania* infection by priming additional MC to adopt an alternative activation phenotype (MC 2), leading to priming of DCs to produce cytokines that favor a Th2-type immune response [37,38,39,40,41,42]. Furthermore, evidence of an extensive release of tryptase and histamine by MC after a natural infection of BALB/c mice with *L. major* and *Ph. duboscqi* was observed by Sanchez-Garcia (2018) [32]. Taken together, the results of our study suggests that MCs possibly play a facilitating role during the early inflammatory events after *L. mexicana* and vector salivary gland proteins enter the host. The dynamics established by the interaction between neutrophils and MCs that facilitate *L. mexicana* infections, is further influenced by hormones.

Throughout evolution, parasites have developed strategies to evade the host immune system and exploit it for their benefit [43]. *L. mexicana* developed a trans-regulation control of the host immune system, as evidenced by the fact that *L. mexicana* promastigotes show increased virulence and infection capacity in the presence of the male hormone dihydrotestosterone [26]. Furthermore, histamine release by MC is dose and gender-dependent [44]. Our results now show that the severity of *L. mexicana* infection is influenced by the male sex hormones that regulate MC activation, promoting rapid neutrophil infiltration and the early release of inflammatory mediators, such as TNF-α, tryptase, and histamine. Thus, hormones are crucial during the development of *L. mexicana* infections in the male host, where the infection is more severe than in females [7].

The slight MC increase in the ear of orchiectomized mice as compared to that of sham mice seems to be related to the absence of male sexual hormones. This observation is in accordance with the literature, where a significant enhancement of MC numbers was reported in orchiectomized male mice, reaching similar numbers as those reported for females [45,46]. Furthermore, the transient mastocytosis in both sham and gonadectomized mice after the stimuli with *L. mexicana* and salivary gland lysates may be related to the antigenic challenge, since this transient mastocytosis was also observed in sham male BALB/c mice after a natural sand fly bite [32].

Sex steroids are involved in *Leishmania* infections [7,15,16,18,23,24,25,26,33], yet the effect of testosterone on the inflammatory response of MCs during *Leishmania* infections has not been analyzed. To address this, we orchiectomized mice to evaluate whether suppression of androgens (mainly testosterone) modified the inflammatory response of MCs to the infection of *Leishmania mexicana* in the presence of sand fly saliva lysates. Orchiectomy is a common technical procedure to evaluate physiological functions of male sex steroids, since other steroids such as estrogens are only produced in low quantities. Testosterone is the androgen in the testes that is responsible for spermatogenesis and whose absence leads to male infertility [47,48]. Previous studies showed that the absence of testosterone in castrated male mice decreased parasite loads in *Leishmania major* infections, whereas the castration of females led to enhanced parasite numbers in the lesions [47,48]. These studies provided the first evidence showing that the deprivation of testosterone and its metabolites by orchiectomy can alter immune mechanisms against *Leishmania*. 

We now show that *L. mexicana* infections in the presence of salivary-gland lysates in orchiectomized mice leads to delayed mast-cell degranulation and diminished release of inflammatory mediators during the early phase of the infection, the combination of which leads to smaller cutaneous ulcers and lower parasite load during the chronic phase of experimental leishmaniasis. Taken together, our data now link the MC response in leishmaniasis to male sexual hormones, which sheds further light on the possible cause of the enhanced male susceptibility to leishmaniasis.

## 4. Materials and Methods

### 4.1. Animals and Experimental Groups

This study used 4-week-old male BALB/c mice that had been bred at the animal facility of the Unidad de Investigación en Medicina Experimental, Medical Faculty, UNAM. All experiments were done following the National Guidelines for animal health NOM-062-ZOO-1999 and were approved by the Internal Ethics Committee for the Care and Use of Laboratory Animals of the Faculty of Medicine, UNAM, with registration number 063-2020/022-CIC-2020. The animals were housed individually in a controlled temperature (22–24 °C) and 12:12 light–dark conditions, receiving a sterilized rodent diet and water ad libitum. The animals were organized into 6 groups of 5 animals each: (1) intact control, (2) infected control, (3) sham intact, (4) sham infected, (5) orchiectomized intact, and (6) orchiectomized infected. Orchiectomized male mice acquired the following characteristics: smooth hair, gained weight, and became less aggressive. At necropsy, animals that presented reminiscence of testes were discarded.

### 4.2. Surgical Procedure

Orchiectomy was performed using a mix of ketamine (0.25%) and xylazine (0.4%) for anesthesia. A small incision was produced in the scrotum, the underlying muscle was cut, and the testes were extruded to the lower abdomen, ligated, and removed. For the sham-operated group, the testes were reinserted and nonligated. Mice were operated under a daylight lamp to keep them warm and monitored daily until staples had self-removed.

### 4.3. Parasite Culture

*L. mexicana* amastigotes were isolated from footpad lesions of infected BALB/c mice, as previously described [23,24]. Promastigotes were obtained by culturing isolated amastigotes at 26 °C in culture medium 199, pH 7.2, supplemented with 10% heat-inactivated fetal bovine serum (FBS), 100 U/mL penicillin G, 100 µg/mL streptomycin, and 2 mM L-glutamine (all from Gibco-Life Technologies, Grand Island, NY, USA) and subpassaged during the logarithmic growth phase (days 3–4 of culture). For in vitro and in vivo infections, promastigotes at the stationary growth phase (day 5 of culture) were used. In this phase, most promastigotes transformed into highly infectious metacyclic promastigotes. All promastigotes were cultured for no more than four in vitro subpassages.

### 4.4. Salivary Gland Collection

Wild female sand flies were caught in Cunduacan, Tabasco, located in the southeast of Mexico, near Grijalva River and Chontalpa subregion. The salivary glands of sand-fly species *Bichromomyia olmeca olmeca* (females) were obtained between 4 and 8 h after collection. Flies were immobilized in cold PBS for about 2 min. The dissected salivary glands were collected in phosphate-buffered saline (PBS) pH 7.2 and stored at −80 °C in batches per collecting day (5 pairs per batch). Lysates of the salivary glands were obtained by sonication and freeze–thaw cycles, and centrifuged at 9900× *g* for 1 min. The supernatant was collected and immediately used. Protein concentration was assessed by DC (DC protein Assay BIORAD 5000002). Average vector salivary-gland protein concentration was 0.66 ± 0.09 µg/µL, corresponding to the pair of salivary glands of each sand fly.

### 4.5. Infection Procedure

The infection procedure was performed as previously described by Dantas et al. (2009) [31]. Briefly, 0.06 µg/µL salivary proteins combined with 100 purified viable metacyclic *L. mexicana* promastigotes were injected into the dermis of both ears of each mouse. The evolution of the infection was monitored at 30 min, and 8, 24, 48, and 72 h. To follow the chronic infection, we measured the size of the ulcer every week and recorded its appearance in photos for 4 and 8 weeks. The average size of the ulcer was reported.

### 4.6. Histology and Immunohistochemistry

The ears were gently flattened onto a piece of thick paper to avoid curling and cut by scissors into three equal fragments (0.7 × 1.6 cm). Ear pieces were fixed in 4% paraformaldehyde in 0.1 M Tris-HCl buffer (pH 7.2) for 24 h. A conventional paraffin-embedded technique was carried out. Tissue sections were stained with hematoxylin–eosin (H&E) or 2% toluidine blue (198161, Sigma Aldrich, Waltham, MA, USA) for histopathological analysis and MC identification, respectively. MCs were identified following the metachromatic principle [49]. TNF-α, histamine, and tryptase were assessed by immunohistochemistry in paraffin-embedded ear tissue sections. The immunohistochemical procedure was as follows: tissue sections were dewaxed with xylene, rehydrated with 0.1 M Tris-HCl buffer (pH 7.2), and transferred to plastic Coplin staining jars containing 0.1 M citrate buffer (pH 6.0) for antigen retrieval. Slides in the Coplin jar were then heated in a pressure cooker for 20 min at 120 °C followed by 10 min at 100 °C. Slides were cooled in the jar at room temperature (RT) for 15 min and then transferred to 0.1 M Tris-HCl buffer (pH 7.2) until needed. After antigen retrieval, endogenous peroxidase was inhibited by incubation for 30 min at RT with 3% hydrogen peroxide diluted in methanol. To reduce nonspecific background staining, slides were then incubated for 1 h at RT in a solution containing 0.1 M Tris-HCl buffer (pH 7.2), 2% BSA, and 0.01% Triton X-100. Slides were incubated overnight at 4 °C with specific primary antibodies anti-TNF- α (1:50 antimouse N-19 sc 1350, Santa Cruz, CA, USA), antitryptase (1:100 Mast Cell Tryptase; antirabbit FL-275, Santa Cruz Biotech, CA, USA), or antihistamine (1:100 antirabbit ab78335 Abcam, Cambridge, UK). After three washes, slides were incubated for 30 min. with biotinylated secondary antibodies, either with antimouse IgG (diluted 1:50) (Jackson Immuno Research Laboratories cat # 115065003) or with antirabbit IgG (diluted 1:50) (Sigma, Waltham, MA, USA) for 1 h at RT. The avidin–biotin–HRP complex and 3,3′-diaminobenzidine were used according to the manufacturer’s instructions (Biocare Medical 901-DB801-010611). Lastly, tissue sections were counterstained with hematoxylin (Sigma HHS16) for 1 min. Only dark brown cells with a visible nucleus and cytological features of MCs were identified as positively stained mast cells. Control tissue sections were processed in the same manner, but primary antibodies were omitted.

### 4.7. Statistics

Nonparametric ordinary one-way ANOVA and multiple comparisons were used to test the statistical significance between groups. Significance was considered at *p* < 0.001. Tests were run by using GRAPH PAD PRISM 8 software.

## Figures and Tables

**Table 1:** Comparison between orchiectomized and sham mast-cell numbers in lesions throughout different time points.

Unpaired Test (*t*-Test)					
Treatments			*p* Value	*p* Value Summary	Significantly Different (*p* < 0.05)
30 min	vs.	30Or	0.0001	***	Yes
8SH	vs.	8Or	0.0001	***	Yes
24SH	vs.	24Or	0.0004	***	Yes
48SH	vs.	48Or	0.0169	*	Yes
72SH	vs.	72Or	0.1369	ns	No

Differences between sham-operated and gonadectomized mice (* *p* ≤ 0.05, *** *p* ˂ 0.001).

## Data Availability

All results were generated and are included in this study.
